# Arsenic in Drinking Water and Incidences of Leukemia and Lymphoma: Implication for Its Dual Effects in Carcinogenicity

**DOI:** 10.3389/fpubh.2022.863882

**Published:** 2022-04-29

**Authors:** Ming-Hsien Lin, Chung-Yi Li, Ya-Yun Cheng, How-Ran Guo

**Affiliations:** ^1^Division of Hematology and Oncology, Department of Internal Medicine, An Nan Hospital, China Medical University, Tainan, Taiwan; ^2^Department of Environmental and Occupational Health, College of Medicine, National Cheng Kung University, Tainan, Taiwan; ^3^Department of Public Health, College of Medicine, National Cheng Kung University, Tainan, Taiwan; ^4^Department of Environmental Health, T.H. Chan School of Public Health, Harvard University, Boston, MA, United States; ^5^Department of Occupational and Environmental Medicine, National Cheng Kung University Hospital, Tainan, Taiwan

**Keywords:** arsenic, incidence, lymphoma, leukemia, carcinogenesis, drinking water

## Abstract

Arsenic in drinking water has been recognized as carcinogenic to humans and can cause solid cancers of lung, urinary bladder, and skin. Positive associations have also been reported between arsenic ingestion and cancers of kidney, liver and prostate. Nevertheless, arsenic trioxide has been used successfully in the treatment of acute promyelocytic leukemia. Therefore, arsenic might play different roles in the carcinogenesis of solid cancers and hematologic malignancies. The relationship between arsenic in drinking water and the incidences of hematologic malignancies has not been fully investigated. We established a cohort of Taiwanese population and assorted 319 townships of Taiwan into two exposure categories using 0.05 mg/L as the cutoff. Then, we linked these data to the Taiwan Cancer Registry and computed standardized incidence ratios (SIRs) of lymphoma and leukemia by sex, exposure category and time period. The trend of changes in the SIRs over time was assessed, from 1981–1990 to 1991–2000 and then to 2001–2010. We found that in both lymphoma and leukemia, the higher exposure category was associated with lower SIRs in both men and women. In terms of time trends, the SIRs in both lymphoma and leukemia showed increasing trends in both sexes, while exposure to arsenic in drinking water decreased over time. The arsenic level in drinking water was negatively associated with the incidences of lymphoma and leukemia in both men and women. This study supports the dual effects of arsenic on carcinogenesis, with a potential protective effect against hematologic malignancies.

## Introduction

Arsenic is a naturally occurring metalloid element common in the earth's crust and often transported by water, so it is widely distributed in natural environments (1). It was once considered an essential trace element for the human body, which led to the hypothesis that arsenic deficiency may be related to the occurrence of cancer (2–4). However, in the process of evolution, humans do not evolve the ability to adapt to or even utilize arsenic like some other creatures (5, 6). Furthermore, since the late 1970s, inorganic arsenic compounds have been considered as carcinogens of skin and lung cancers in humans (7). Thereafter, arsenic in drinking water has been classified as group 1 carcinogen by the International Agency for Research on Cancer (IARC) (8).

Almost all inorganic arsenic compounds are likely to be carcinogenic. Proposed mechanisms of arsenic-induced carcinogenesis include, but not limited to, biotransformation as a toxicity activation mechanism, generation of reactive oxygen species, and epigenetic mechanisms such as changes in DNA methylation patterns, histone modifications, and altered expression of microRNAs (9). They can cause solid cancers of the lung (10, 11), urinary bladder (12), and skin (13), and are also positively correlated with cancers of the liver, prostate, and kidney (14, 15), particularly transitional cell carcinoma of the renal pelvis (12). However, previous studies did not work out a consistent association between arsenic exposure and the risk of hematologic malignancies. A study collected blood samples of 2,000 confirmed cancer patients in an endemic area of arsenic exposure in India and found that arsenic levels in carcinoma patients were higher as compared to patients with sarcomas, lymphomas, and leukemia (16). A study in Idaho, USA, where arsenic has been found to exceed 10 μg/L in ground water in some counties, found that exposure to arsenic in ground water was not associated with cancer incidence when adjusting for salient variables (17). Whereas, there seemed to be a dose-response relationship between the arsenic level and incidence of non-Hodgkin lymphoma in males, females living in the low arsenic counties had higher incidence than those who living in the intermediate arsenic counties or the high arsenic counties. However, the differences did not reach statistical significance.

On the other hand, arsenicals have been used as medicinal therapeutics since ancient times. Fowler solution (1% potassium arsenite, KAsO_2_) was first described in 1845 and used to treat anemia, and it was also the first chemotherapeutic agent used in the late nineteenth century for the treatment of leukemia. In 1931, Forkner and Scott rediscovered Fowler solution to treat chronic myeloid leukemia (CML), and it remained the treatment of choice until busulphan was introduced in 1953 (18, 19). In the 1990s, the Shanghai Institute of Hematology reported the efficacy of arsenic trioxide in treating acute promyelocytic leukemia (APL) (20, 21). It was considered to have dose-dependent dual effects on APL cells: inducing preferentially apoptosis at relatively high concentrations (0.5–2.0 mmol/L) and partial differentiation at low concentrations (0.1–0.5 mmol/L) (22, 23).

There are increasing arguments supporting that epigenetic regulation plays an important role in carcinogenesis, including the tissue organization field theory (24) and the six-step multi-sequence hypothesis (25). Differentiation through histone modifications has been recognized as a fundamental process of epigenetic regulation (26); and arsenic toxicity also has an emerging role in epigenetic dysregulation (27). Therefore, the dual effects of arsenic toxicity could play different roles in the carcinogenesis of solid cancers and hematologic malignancies. That is, at the appropriate exposure concentration and action duration, arsenic may reverse the carcinogenesis of hematologic malignancies, probably by the differentiation effect through histone modifications. While chromosome anomaly plays a pathogenic role in the origin and progression of hematologic malignancies, cytotoxicity with consequent regenerative proliferation may be the most likely mode of action for many carcinomas. Evidende supporting this has been demonstrated for inorganic arsenic, specifically in the DMA^v^ rat bladder cancer model (28).

According to the annual reports of Taiwan Cancer Registry, in 1995, the age-adjusted annual incidence rates of non-Hodgkin's lymphoma (NHL) of men and women were 4.77/10^5^ and 3.42/10^5^, and the rates were 4.12/10^5^ and 2.87/10^5^ for leukemia of men and women, respectively. In 2010, these figures of NHL of men and women had increased to 8.13/10^5^ and 5.97/10^5^, and 7.91/10^5^ and 5.48/10^5^ for leukemia of men and women, respectively. Furthermore, the mortality rates of lymphoma and leukemia have always been among the top ten major cancers in Taiwan, where high levels of arsenic have been found in the drinking water in many townships since more than half a century ago.

To fill the data gap in the relationship between arsenic and hematologic malignancies, we conducted a study spanning 30 years to evaluate the associations between the exposure of arsenic in drinking water and incidences of lymphoma and leukemia. An important issue taken into consideration is the fact that a tap water supply system had been implemented in the 1960s in the endemic areas of arsenic exposure from drinking water (29). Changes in the incidences should provide insights to the causal relationship between arsenic and the diseases.

## Materials and Methods

### Study Population

We established a cohort of Taiwanese population based on information from the “Taiwan-Fukien Demographic Fact Book.” Taiwan's Ministry of the Interior tabulates and compiles the population data produced from household registration into this annual publication, which contains absolute figures of both static and dynamic population data as well as relative rates and ratios calculated in accordance with the international standards. This cohort started from January 1, 1901 and included all Taiwanese born before January 1, 1961. Therefore, members of this cohort were over 20 years old on January 1, 1981. The cohort size counted 20,461,396 subjects, of which 10,564,150 were men. Each person was followed from January 1, 1981, to the date of diagnosis of hematologic malignancies (lymphoma or leukemia), emigration, death, or December 31, 2010. We excluded patients who were diagnosed with lymphoma or leukemia at an age younger than 20 years old. The exclusion is to take into consideration the facts that genetic factors play a major role in the development of cancers in young children and that a tap water supply system was implemented in the 1960s in the endemic areas of arsenic exposure from drinking water. We also excluded patients who were 80 years of age or older at diagnosis due to the extremely small number of cases.

### Assessment of Arsenic in Drinking Water

The arsenic levels in drinking water were assessed on the basis of a census survey of drinking water conducted by the Taiwan Provincial Institute of Environmental Sanitation (30). The survey measured arsenic concentrations in more than 80,000 wells mostly between 1974 and 1976, and the results were available for 311 townships. The reports of the survey classified arsenic concentrations in the drinking water into three levels: “ <0.05 mg/L,” “0.05–0.35 mg/L,” and “>0.35 mg/L.” The cutoff 0.05 mg/L is the regulatory standard at the time of survey, and the cutoff 0.35 ml/L was determined by the survey team according to the severity of health effects. We further reclassified the three concentration levels into two exposure categories as “low” (<0.05 mg/L) and “high” (≥0.05 mg/L) because the numbers of cases observed in the townships with arsenic levels >0.35 mg/L were small. The database center where we performed our analyses does not allow obtaining information on small groups of individuals due to confidentiality. Besides, small case numbers cannot generate stable risk estimates.

### Identification of Cases

We collected data on incident cancer cases from the Taiwan Cancer Registry, including sex, birth year, age at diagnosis, pathologic diagnoses coded according to the International Classification of Diseases for Oncology, third edition (ICD-O-3), and the township of residence of each patient. We defined those with ICD-O-3 codes from 959 to 972 as lymphoma, and 980 to 998 as leukemia. The registry was established in 1979 but was not fully operated in the first several years. Therefore, we aggregated data for the years 1981 through 2010, about half a century after the construction of the tap water system in the endemic areas, and stratified by sex, age (in 10-year group), year (in 10-year period), exposure category, and cancer type. Data on the population of each township were drawn from the annual “Taiwan-Fukien Demographic Fact Book.”

### Statistical Analysis

We calculated sex-age-year specific cancer incidence rates in the townships of the “low” exposure category. Then, the sex-age-year specific incidence rates were used as the reference to estimate the expected number of cases in each township in the “high” exposure category. The standardized incidence ratios (SIRs) were computed by dividing the observed number of cases in a given township by the sex-age-year specific expected number of cases for the same township. To assess the trends of changes in the SIRs over time, we divided the study period into three decades: 1981–1990, 1991–2000, and 2001–2010.

Throughout the study, all personal identification information was removed, and patients remained anonymous. The study protocol was reviewed and approved by the Institutional Review Board of National Cheng Kung University Hospital (A-EX-105-021, A-EX-108-045), and the research had been conducted according to the principles of Declaration of Helsinki. We performed all statistical analyses using the Statistical Analysis Software Version 9.4 (SAS Inc., Raleigh, NC, USA). Both the 95% confidence limits and the statistical significance threshold had been considered, and all statistical tests were performed at the two-tailed significant level of 0.05.

## Results

We identified 3,863 lymphoma and 2,889 leukemia cases in the period 1981–1990; 9,046 lymphoma and 5,313 leukemia cases in the period 1991–2000; and 14,639 lymphoma and 10,299 leukemia cases in the period 2001–2010. The number of cases generally increased over the three consecutive 10-year time periods, and there were more male cases than female cases of both lymphoma and leukemia in both exposure categories and in each time period (Table 1).

**Table 1:** The number of incident cases of lymphoma and leukemia by sex and exposure category in three consecutive 10-year time periods.

	**1981–1990**		**1991–2000**		**2001–2010**	
	**Male**	**Female**	**Male**	**Female**	**Male**	**Female**
Lymphoma
Low	1,842	1,031	3,995	2,526	5,873	4,528
High	628	362	1,485	1,040	2,474	1,764
Leukemia
Low	1,293	815	2,249	1,523	4,235	2,935
High	485	296	870	671	1,855	1,274

For lymphoma, the SIRs indicated a lower risk associated with the high exposure category in both men and women in all the three time periods. The reductions in the risk reached statistical significance except for men in 2001–2010. We also found that the SIRs in men had a consistent increasing trend over the three consecutive 10-year periods (*p* < 0.001). This indicated a decreasing trend in the negative association over time. In women, the reductions in the risk associated with the high exposure category reached statistical significance only in 1981–1990, but the SIRs in 1991–2000 and 2001–2010 (both were 0.93) did not reach statistical significance. Consequently, the SIRs in women did not have a significant trend over time (*p* = 0.131).

For leukemia, the high exposure category was associated with a lower risk in both sexes in 1981–1990 and in men only in 1991–2000. In terms of the time trend, we found that only the SIRs in men had a consistent increasing trend over the three consecutive 10-year periods (*p* = 0.007). This indicated a decreasing trend in the negative association over time. In women, there was also a consistent increasing trend in SIRs over the three consecutive 10-year periods, but the changes were small (*p* = 0.066) (Table 2).

**Table 2:** The standardized incidence ratio (SIR) and its 95% confidence interval (CI) associated with the high exposure category relative to the low exposure category by sex and time period.

	**1981–1990**			**1991–2000**			**2001–2010**		
	**O**	**E**	**SIR (95% CI)**	**O**	**E**	**SIR (95% CI)**	**O**	**E**	**SIR (95% CI)**
Leukemia
Male	485	551	0.88 (0.80–0.96)	870	949	0.92 (0.86–0.98)	1,855	1,793	1.03 (0.99–1.08)
Female	296	341	0.87 (0.77–0.97)	671	657	1.02 (0.94–1.10)	1,274	1,220	1.04 (0.99–1.10)
Lymphoma
Male	628	787	0.80 (0.74–0.86)	1,485	1,695	0.88 (0.83–0.92)	2,474	2,487	0.99 (0.96–1.03)
Female	362	453	0.80 (0.72–0.88)	1,040	1,116	0.93 (0.88–0.99)	1,764	1,898	0.93 (0.89–0.97)

*O, observed number of cases; E, expected number of cases*.

## Discussion

The current study observed a lower risk of lymphoma associated with the high exposure category in both sexes across all the three consecutive 10-year time periods, and the reductions reached statistical significance in both sexes in 1981–1990 and only men in 1991–2000. The reductions did not reach statistical significance in both sexes in 2001–2010. For leukemia, a lower risk associated with the high exposure category was also observed in both sexes in 1981–1990 and only men in 1991–2000. In general, the SIRs support a negative association between the arsenic exposure category and the incidences of hematologic malignancies. In both lymphoma and leukemia, there was an increasing trend in the SIRs over time in both sexes, except for lymphoma in women from 1991–2000 to 2001–2010.

Because a tap water supply system was implemented in the 1960s in the endemic areas of arsenic exposure from drinking water and artesian well water was no longer used for drinking after the mid-1970s (29), the increasing trends of time sequence observed in SIRs indicated that the earlier the time period apart from the time of exposure cessation, the more obvious the effect of decreasing SIRs of both lymphoma and leukemia. The time trends further support a negative association between the arsenic exposure category and the incidence of hematologic malignancies.

Most previous studies on the associations between arsenic exposure and cancer focused on the mortality risk of solid cancers, and few studies mentioned hematologic malignancies. Through a review of literature, we identified a handful of epidemiological studies that provided quantitative risk estimates of hematologic malignancies in adults in association with arsenic in drinking water, including three in Taiwan (Table 3).

**Table 3:** Previous studies of the association between arsenic in drinking water and hematologic malignancies.

**References**	**Disease (ICD−9 code)**	**Outcome**	**Risk estimate (95% CI)**
Chen (28)	Leukemia (204–208)	Mortality	**Standardized mortality ratio**
			Male: 1.42 (1.00–1.84)
			Female: 0.90 (0.53–127)
Wu (29)	Leukemia (204–208)	Mortality	**Age-adjusted mortality rate**
			Male: no dose-response relationship
			Female: no dose-response relationship
Tsai (30)		Mortality	**Standardized mortality ratio**
	Lymphoma (200–202)		Male: 1.42 (1.07–1.84)
			Female: 1.43 (1.00–1.99)
	Leukemia (204–208)		Male: 1.34 (1.04–1.70)
			Female: 1.05 (0.75–1.43)
Hinwood (31)		Incidence	**Standardized incidence ratio**
	Other lymphoma and leukemia (200–205.0)		Not significant
	Chronic myeloid leukemia (205.1)		1.54 (1.13–2.10)
García-Esquinas (32)	Lymphatic and hematopoietic tissue (200–208)	Mortality	**Hazard ratio**
			0.46 (0.22–0.96)

As compared with the general population in Taiwan, Chen et al. found that the standardized mortality ratio was significantly higher in the black-foot disease endemic area, where the arsenic levels in drinking water were generally high, for cancers of the bladder, kidney, skin, lung, liver, and colon, but the mortality rate of leukemia was about the same (31). Using 0.30 and 0.60 mg/L as the cutoffs, Wu et al. further declared that a significant dose-response relationship was observed between arsenic levels in well water and the age-adjusted mortality rates of cancers of the bladder, kidney, skin, and lung in both men and women in the black-foot disease endemic area. However, such a relationship was not observed in the mortality rate for leukemia (32). Tsai et al. also studied the mortality for certain disease in the black-foot disease endemic area and discovered a “high-but-marginal” [with a lower limit of the 95% confidence interval (CI) close to 1] mortality for leukemia in men only (33). However, the study treated the population in the black-foot disease endemic area as a whole and did not divide them according to the exposure level. Overall, the previous studies in Taiwan did not observe the negative association between arsenic exposure level and hematologic malignancies, but only one of them categorized the exposed study population according to arsenic level in drinking water. However, that study used 0.30 mg/L as the lowest cutoff, which is six times of that used in our study.

Hinwood et al. investigated the cancer incidence in relation to high environmental arsenic concentrations and observed elevated SIR for CML but not for other lymphoma and leukemia. However, the increased SIR of CML was only significant in the high water/high soil category, resulting in a lack of significant dose response relationship (34). In addition, among the cancers that “previously associated with arsenic exposure” (namely cancers of the nasal cavity, prostate, liver, lung, bladder, and kidney, determined according to the published literature), only prostate cancer showed a small but significant excess; on the contrary, of the other cancers examined that are not “previously associated with arsenic exposure,” melanoma and breast cancer were elevated and statistically significant, besides CML. These observations indicated that the exposure categorization could not predict accurately the cancer risk associated with arsenic exposure, and thus the associations observed between arsenic ingestion and CML might not be a causal association.

García-Esquinas et al. evaluated the association between low-moderate arsenic exposure in drinking water and cancer mortality in about four thousand American Indians and estimated inorganic arsenic exposure on the basis of the sum of inorganic and methylated species in urine (35). They found that arsenic exposure was associated with decreased mortality from lymphatic and hematopoietic malignancies. Nonetheless, due to the small number of cases, it was not feasible to further evaluate the association between arsenic and lymphoma or leukemia separately. Overall, the results are compatible with those in the current study.

As artesian wells were the sources of drinking water with high arsenic levels, a series of studies have been conducted to evaluate the changes in the risks of cancers after the establishment of the tap water system. According to the IARC, arsenic causes cancer of the lung, urinary bladder, and skin, and a positive association has been observed between exposure to arsenic and cancer of the kidney, liver, and prostate (14). Among the three types of cancers of which arsenic is regarded as a cause, studies have been conducted on lung and urinary bladder cancers to evaluate the time trends in Taiwan, and the results showed that the risks of both decreased after the establishment of the tap water system (29, 36).

The establishment of the tap water system also led to a reduction in the risk of prostate cancer in the endemic area in Taiwan (37), and the IARC argued that the existing evidence suggests the possibility of a causal associations between arsenic in drinking water and prostate cancer, particularly in Taiwan (14). For liver cancer, the risk reduction was observed in females, but not in males (38). As argued by the IARC, although data from epidemiological studies strongly suggest a causal association between arsenic in drinking water and liver cancer, hepatitis B is a major confounding factor in studies in Taiwan (14), which may contribute to the difference observed between the two sexes in that study.

Overall, the changes in the risks of cancers in Taiwan after the establishment of the tap water system were compatible with the conclusion made by the IARC regarding the types of cancer associated with arsenic exposure from drinking water. Therefore, the increasing time trend in the SIRs (indicating a decreasing trend in the reduction of the risk) of lymphoma and leukemia observed in the current study further supports a negative association between the arsenic exposure category and the risk of hematologic malignancies.

A major limitation of the current study is the use of ecological data, and therefore, the results of our analyses might be affected by “ecological fallacy” and also other common limitations of ecological study design, such as misclassifications of exposure, inability to address exposure duration, and biases introduced by effects of population mobility. To better define the relationships, further studies with exposure data of individuals are needed. In addition, different subtypes of lymphomas and leukemias might have different degrees of association with arsenic exposure. Because the numbers of cases of lymphomas and leukemias were not large enough to perform further analyses for each subtype, we had to lump all lymphomas together and all leukemias together. Further studies with more cases are needed to identify specific subtypes of cancer and characteristics of individuals, such as age, for which the associations with arsenic exposure are particularly higher.

Some factors other than genetic makeup can also contribute to the development of lymphoma and leukemia, and thus may become confounding factors in the current study, including virus, radiation, and smoking. However, Taiwan is not an endemic area for those viruses-associated lymphoma and leukemia, such as the Epstein-Barr virus associated Burkitt's lymphoma and the human T-cell leukemia virus type-1 associated adult T-cell leukemia (39, 40). As for radiation, Taiwan does not have any endemic area of radon exposure, and according to the Radiation Monitoring Center of Taiwan's Atomic Energy Council, none of the environmental measurements has exceeded the safety limits (41). Although we were unable to adjust for the effects of smoking directly, the observations on women were unlikely to be substantially influenced by the unmeasured smoking because the smoking rate among Taiwanese women is very low, 3.2% in 1992 and 4.1% in 2010 (42). Our analyses showed similar findings in women and men for both lymphoma and leukemia, which provides reassurance that the confounding effects of smoking on the association observed in men, were likely to be small, if any.

Nonetheless, the current study has some advantage. Most of the previous studies focused on arsenic exposure and its risks of cancers were based on mortality. However, mortality is affected by the disease fatality rate, while incidence is the exact measure of disease risk. Therefore, to assess the risk of exposure to disease, incidence is more appropriate than mortality, and the current study is based on incidence data. Another advantage is that we not only compared the incidence between populations with different exposure levels, but also assessed the longitudinal trend of time. In connection with the establishment of the tap water system, observations on the time trend provide further support to a causal relationship. Furthermore, we included a very large population and therefore can obtained stable estimates on relatively rare diseases such as lymphoma and leukemia.

The observation of a negative association between the exposure category and incidence of hematologic malignancies in our study support and elucidate the dual effects of arsenic, which may promote epigenetic differentiation of carcinogenesis through histone modifications in hematologic malignancies. Larger, carefully designed epidemiologic studies will be required to more comprehensively examine the presence and consequence of arsenic-induced alterations in populations affected by arsenic contamination. Nonetheless, the results of the current study support the therapeutic usages of arsenic on hematologic malignancies to a larger extent.

## Data Availability Statement

Publicly available datasets were analyzed in this study. The data can be found here: Taiwan Cancer Registry.

## Ethics Statement

The studies involving human participants were reviewed and approved by Institutional Review Board of National Cheng Kung University Hospital (A-EX-105-021, A-EX-108-045). Written informed consent from the participants' legal guardian/next of kin was not required to participate in this study in accordance with the national legislation and the institutional requirements.

## Author Contributions

M-HL and H-RG conceived and designed the study, analyzed the data, and wrote the manuscript. C-YL and Y-YC provided editorial support. All authors approved the manuscript.

## Funding

This study was supported by a research grant ANHRF105-04 from the Tainan Municipal An-Nan Hospital-China Medical University.

## Conflict of Interest

The authors declare that the research was conducted in the absence of any commercial or financial relationships that could be construed as a potential conflict of interest.

## Publisher's Note

All claims expressed in this article are solely those of the authors and do not necessarily represent those of their affiliated organizations, or those of the publisher, the editors and the reviewers. Any product that may be evaluated in this article, or claim that may be made by its manufacturer, is not guaranteed or endorsed by the publisher.
