# Corrigendum: Arsenic in drinking water and incidences of leukemia and lymphoma: Implication for its dual effects in carcinogenicity

**DOI:** 10.3389/fpubh.2022.965779

**Published:** 2022-12-16

**Authors:** Ming-Hsien Lin, Chung-Yi Li, Ya-Yun Cheng, How-Ran Guo

**Affiliations:** ^1^Division of Hematology and Oncology, Department of Internal Medicine, An Nan Hospital, China Medical University, Tainan, Taiwan; ^2^Department of Environmental and Occupational Health, College of Medicine, National Cheng Kung University, Tainan, Taiwan; ^3^Department of Public Health, College of Medicine, National Cheng Kung University, Tainan, Taiwan; ^4^Department of Environmental Health, T.H. Chan School of Public Health, Harvard University, Boston, MA, United States; ^5^Department of Occupational and Environmental Medicine, National Cheng Kung University Hospital, Tainan, Taiwan

**Keywords:** arsenic, incidence, lymphoma, leukemia, carcinogenesis, drinking water

In the published article, there was an error in part of the title. Instead of “Implication for its dural effects in carcinogenicity” it should read “Implication for its dual effects in carcinogenicity.” The corrected full title appears below:

“Arsenic in drinking water and incidences of leukemia and lymphoma: Implication for its dual effects in carcinogenicity”

A correction has been made to the **Abstract**. The sentence previously stated:

“In terms of time trends, the SIRs in both lymphoma and leukemia showed decreasing trends in both sexes, while exposure to arsenic in drinking water also decreased over time.”

The corrected sentence appears below:

“In terms of time trends, the SIRs in both lymphoma and leukemia showed increasing trends in both sexes, while exposure to arsenic in drinking water decreased over time.”

The authors apologize for the errors and state that this does not change the scientific conclusions of the article in any way. The original article has been updated.

